# A Novel Immune-Related Prognostic Signature in Head and Neck Squamous Cell Carcinoma

**DOI:** 10.3389/fgene.2021.570336

**Published:** 2021-06-18

**Authors:** Yi Zhang, Ping Chen, Qiang Zhou, Hongyan Wang, Qingquan Hua, Jie Wang, Hongliang Zhong

**Affiliations:** ^1^Department of Otolaryngology-Head and Neck Surgery, Beijing Chaoyang Hospital, Capital Medical University, Beijing, China; ^2^Department of Urology, Zhongnan Hospital of Wuhan University, Wuhan, China; ^3^Department of Otolaryngology-Head and Neck Surgery, Renmin Hospital of Wuhan University, Wuhan, China; ^4^Department of Neurosurgery, Beijing Chaoyang Hospital, Capital Medical University, Beijing, China

**Keywords:** head and neck squamous cell carcinoma, immunogenomic landscape, prognosis, the cancer genome atlas, prognostic signature

## Abstract

The immune response within the tumor microenvironment plays a key role in tumorigenesis and determines the clinical outcomes of head and neck squamous cell carcinoma (HNSCC). However, to date, very limited robust and reliable immunological biomarkers have been developed that are capable of estimating prognosis in HNSCC patients. In this study, we aimed to identify the effects of novel immune-related gene signatures (IRGs) that can predict HNSCC prognosis. Based on gene expression profiles and clinical data of HNSCC patient cohorts from The Cancer Genome Atlas (TCGA) and Gene Expression Omnibus (GEO) database, a total of 439 highly variable expressed immune-related genes (including 239 upregulated and 200 downregulated genes) were identified by using differential gene expression analysis. Pathway enrichment analysis indicated that these immune-related differentially expressed genes were enriched in inflammatory functions. After process screening in the training TCGA cohort, six immune-related genes (*PLAU*, *STC2*, *TNFRSF4*, *PDGFA*, *DKK1*, and *CHGB*) were significantly associated with overall survival (OS) based on the LASSO Cox regression model. Integrating these genes with clinicopathological features, a multivariable model was built and suggested better performance in determining patients’ OS in the testing cohort, and the independent validation cohort. In conclusion, a well-established model encompassing both immune-related gene signatures and clinicopathological factors would serve as a promising tool for the prognostic prediction of HNSCC.

## Introduction

Head and neck squamous cell carcinoma (HNSCC) is one of the world’s leading causes of morbidity and mortality, accounting for about 4% of all cancers (Riba et al., 2019). In the United States in 2018, it was estimated that about 51,540 patients would be diagnosed with oral and throat cancer, and that 10,030 patients would die from this disease (Siegel et al., 2018). HNSCC accounts for 95% of head and neck cancers and occurs in the mouth, oropharynx, hypopharynx, or throat (Haddad and Shin, 2008). The 5-year survival rate for HNSCC patients is only 63%, mainly because local recurrence or distant metastasis occurs in approximately 80–90% of patients with advanced HNSCC. Although there has been a significant advancement in surgical treatment, radiation therapy, and chemotherapy, there has been no clear improvement in the 5-year survival rate of patients with HNSCC (von Mehren et al., 2018).

The specific cause of head and neck tumors is still unknown currently. Previous studies have shown that smoking is a common risk factor for pharyngeal, laryngeal, throat, and oral, malignancies (Chen et al., 2018; Stanford-Moore et al., 2018). Human papillomavirus (HPV) infection has been shown to have an important relationship with the occurrence of velopharyngeal cancer (Mork et al., 2001; Marur et al., 2010; Nishat et al., 2015). Epstein Barr virus (EBV) infection is one of the main causes of nasopharyngeal carcinoma (Turunen et al., 2017). These studies suggest that the occurrence of HNSCC tumors is a multi-step, and multi-factorial process.

In recent years, an extensive number of publications have suggested that the tumor microenvironment was associated with prognosis in various cancer types (Ferris, 2015; Economopoulou et al., 2016; Chen et al., 2020a). Deeply understanding the immune activity in the tumor microenvironment would provide researchers and clinicians with more accurate prognostic information (Li et al., 2019, 2020a). Many studies reported that tumor-infiltrating immune cells and gene networks of the immune cell were associated with cancer initiation and progression in HNSCC (Na and Choi, 2018). For instance, a recent study identified EGFR and PTGS2 as key nodes in a gene regulatory network related to the immune phenotype in HNSCC (Feng et al., 2020). However, to date, reliable and predictive biomarkers that have been used for identifying HNSCC are limited. Therefore, developing immune-related signatures in HNSCC would assist in understanding the potential prognostic value of the immunogenomic profile and improve the understanding of infiltrating immune cell function in HNSCC. In this study, we sought to employ integrative analysis to develop a novel marker based on cancer immunogenomic profiles, which could predict the prognosis of HNSCC.

## Materials and Methods

### HNSCC Tumor Dataset and Processing

We obtained raw RNA-sequencing data and corresponding clinical information from cases of head and neck squamous cell carcinoma from the TCGA data portal. We also downloaded the microarray dataset GSE65858 from the GEO database. Both mRNA expression profiles and clinical information relating to HNSCC are open-access and publicly available. Therefore, ethical approval by a local ethics committee was not needed for this study. Gene expression data relating to HNSCC in the TCGA related to 502 HNSCC specimens and 44 adjacent non-tumor specimens; GSE65858 featured 270 samples of HNSCC tissue. The IDs for these samples were converted to gene symbols by the corresponding GENCODE files. The “edgeR” R package was used to identify differentially expressed genes (DEGs) for RNA-sequencing data, while the “limma” R package was used to process the gene microarray data. The cut-off criteria for screening DEGs were a | log2 (fold change)| ≥ 1 and a default Benjamini-Hochberg false discovery rate (FDR) < 0.05.

### Functional Enrichment Analysis

To explore the potential biological functions of differentially expressed immune-related genes, we performed functional enrichment analysis utilizing the Metascape web portal (Zhou et al., 2019). Metascape is an automated meta-analysis tool for gene annotation and functional enrichment analysis. This tool also used the MCODE algorithm to infer more biologically interpretable protein-protein interaction analysis through automatically extracted protein complexes embedded in the large network (Bader and Hogue, 2003).

### Establishment of IRGs Signature With LASSO Cox Regression Model

LASSO Cox regression analysis (LASSO, least absolute shrinkage, and selection operator (Tibshirani, 1997)) can achieve shrinkage and variable selection simultaneously by performing the Cox regression model with LASSO penalty (Luo et al., 2019a). This technique was used to establish the most valuable and predictable IRG signature. First, all samples in the TCGA datasets were randomly separated into a training cohort (*n* = 369) and a test cohort (*n* = 123) in a ratio of 3:1 using a randomization method. Univariable Cox analysis was then used to identify IRGs associated with prognosis. LASSO Cox regression analysis was then performed using the “glmnet” tool in the R package to construct a prognostic gene signature of IRGs utilizing survival-related IRGs in a training cohort. We estimated the optimal values of penalty parameter lambda via 10-fold cross-validation (Goeman, 2010). A prognostic IRG signature was then constructed using the gene expression value weighted by the estimated regression coefficient in the LASSO Cox regression model. The risk score of a six-IRG signature was calculated based on the formula below:

$$\text{Risk score}=\sum_{\text{i=1}}^{\text{n}}\left({\text{coef}}_{i}*{\text{Expr}}_{i}\right)$$

in which Expr*i* represents the expression value of the IRGs in the signature for patient I and *coefi* represents the LASSO coefficient of the IRGs.

### Evaluation of the Prognostic Value of the IRG Signature

Patients were assigned into a high-risk group and a low-risk group in accordance with the median cut-off values for the six-IRG signature (Camp et al., 2004). We then performed Kaplan–Meier survival analysis and a log-rank test to assess the association between each IRG and patient survival in the test cohort. We used the GSE65858 dataset as a validation cohort to further evaluate the prognostic value of the six-IRG signature. Additionally, a multivariable Cox regression model with integrated subgroup information was performed to investigate whether the six-IRG signature was an independent predictive marker for HNSCC patients.

### Establishment and Validation of a Predictive Nomogram

A nomogram was used to construct a prognostic scoring system for predicting survival in HNSCC patients. Calibration plots were performed to validate the performance of the nomogram. Decision curve analysis was then used to estimate the clinical utility of the nomogram (Vickers and Elkin, 2006; Kerr et al., 2016). R packages “rms” and “rmda” were used for the establishment and validation of the nomogram.

### Estimating the Correlation of the IRG Signature With Immune Cells Infiltration

TIMER^1^ is an online web database (Li et al., 2016), and includes 10,897 samples across 32 cancer types from TCGA. The TIMER database provides an open platform with which to identify and visualize the abundance of tumor-infiltrating immune cells. The TIMER database can be used to infer the abundance of six subtypes of tumor-infiltrating immune cells to form an expression profile, including B cells, CD4 T cells, CD8 T cells, dendritic cells, macrophages, and neutrophils. We obtained immune infiltrate levels for HNSCC patients according to the TIMER database and then analyzed the correlation between the abundance of tumor-infiltrating immune cells with the expression of the IRG signature.

### Genetic Alteration of IRGs and the Construction of a Gene-Gene Interaction Network

The cBioPortal^2^ for Cancer Genomics is an open platform that can be used to explore, visualize, and analyze cancer genomic data. This includes multi-dimensional cancer genomic data from 32 types of cancer from the TCGA database (Cerami et al., 2012). This tool is available for researchers to investigate the genetic alterations of different samples, genes, and pathways (Luo et al., 2019b). We utilized the cBioPortal database to identify genetic alterations of the six IRGs. Then, we used the maftools package to analyze the mutation status of the six IRGs in HNSCC tumors (Kandoth et al., 2013; Mayakonda et al., 2018). Interaction networks for the six immune-related prognostic genes were then analyzed using the GeneMANIA plugin in Cytoscape (Montojo et al., 2014). They showed the physical, co-expression, and pathway gene-gene interactions of these six IRGs.

### Quantitative Reverse Transcription Polymerase Chain Reaction (qRT-PCR) Assays

Sixteen HNSCCs and corresponding adjacent tissues were obtained from the Renmin Hospital of Wuhan University to validate the expression of the six IRGs, information on the key clinicopathologic features for these samples are detailed in Supplementary Table 1. The study was approved by the Ethics Committee of the Renmin Hospital of Wuhan University and all participants provided written informed consent.

Total RNA samples were extracted from the frozen HNSCC tissues using TRIZOL reagent (Invitrogen, Canada), in accordance with the manufacturer’s guidelines. RNA (1 μg/sample) was reversed-transcribed into cDNA using the PrimeScript RT Reagent Kit (TaKaRa Biotechnology, Dalian, China). SYBR Green Real-time PCR Master Mix-Plus kits (TaKaRa Biotechnology, Dalian, China) were then used to perform triplicate PCR assays to determine the mRNA levels of the target genes. The 2^–Δ*Ct*^ method was used to demonstrate relative mRNA expression (Wang et al., 2016). GAPDH was chosen as the internal reference for qRT-PCR. Supplementary Table 2 shows the primers used in our study.

### Statistical Analysis

All the statistical analyses were performed utilizing R software (version: 3.5.2). All statistical tests were performed by using a *p*-value < 0.05 (two-sided) as the statistically significant threshold. Group comparisons were performed utilizing the *t*-test for continuous variables and χ^2^-test for categorical variables.

## Results

### Identification of Immune-Related Genes and Pathway Analysis

**FIGURE 1:**
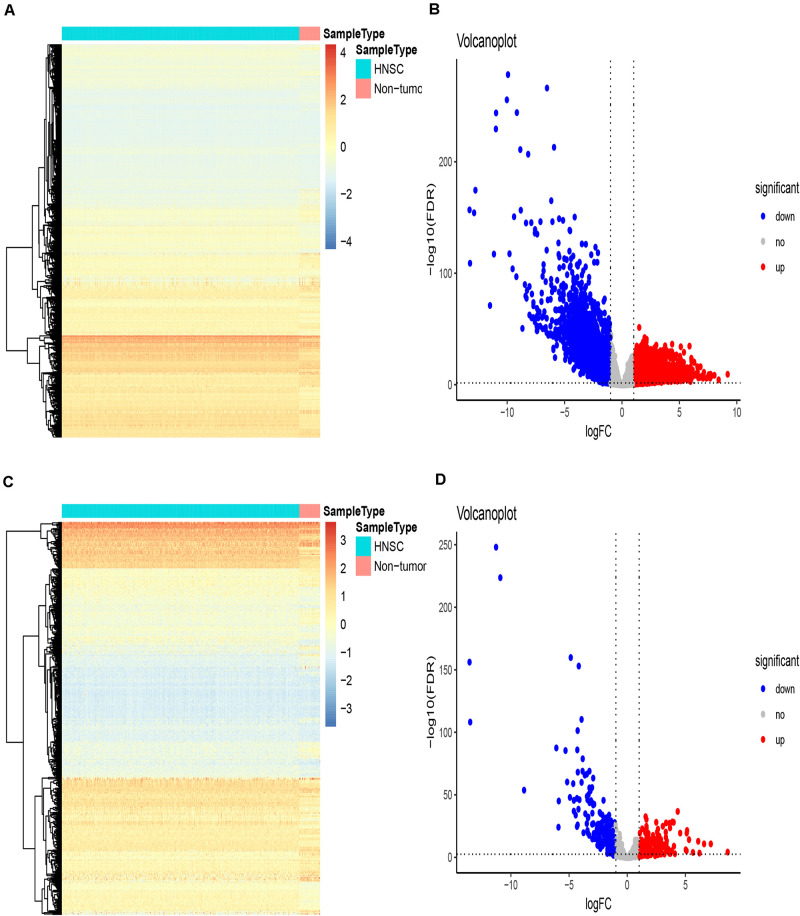
Differentially expressed immune-related genes. **(A–C)** Heatmaps showing differentially expressed genes in the TCGA dataset and differentially expressed immune-related genes (IRGs), respectively, based on the cut-off criteria of | log2 (fold change)| ≥ 1 and the default Benjamini-Hochberg false discovery rate (FDR) < 0.05. The color indicates the level of expression of the gene (red is upregulation, blue is downregulation). Each row represents the expression level of each gene in different samples, and each column represents the expression level of all genes in each sample. The tree diagram shows the results of cluster analysis of different genes from different samples. **(B–D)** Volcano plots showing differentially expressed genes in the TCGA dataset and differentially expressed immune-related genes (IRGs), respectively. The red nodes represent upregulated genes while the green nodes represent downregulated genes.

**FIGURE 2:**
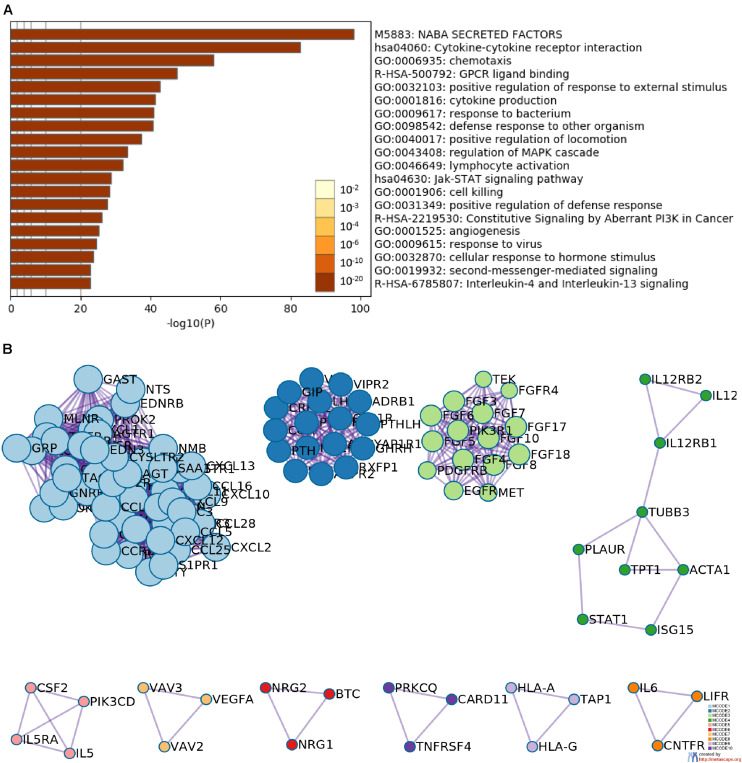
Functional enrichment and network analysis. **(A)** Heatmap of the top 20 functional enrichment terms (including Gene Ontology and pathways); each bar represents a cluster. **(B)** Network analysis revealed the 10 biologically interpretable protein-protein interaction subnetworks identified by the MCODE algorithm.

The gene expression matrix of the TCGA-HNSCC dataset was analyzed to identify DEGs, this matrix consisted of 502 HNSCC samples and 44 adjacent non-tumor samples. A total of 5790 DEGs were identified with | log2FC| > 1 and an FDR < 0.05, including 3532 upregulated genes and 2258 downregulated genes. Then, we obtained IRGs from the Immunology Database and Analysis Portal (Nowis et al., 2005; Bhattacharya et al., 2014); 339 IRGs were differentially expressed, including 139 upregulated genes and 200 downregulated genes. Heatmaps show the unsupervised clustering of all DEGs and IRGs (Figures 1A,C). Volcano plots were used to present significant differences in DEGs and IRGs when compared between HNSCC tumor samples and adjacent non-tumor samples (Figures 1B,D). Protein-protein interaction analysis was then carried out with Metascape to analyze the pathway enrichment and interaction network of differentially expressed IRGs, analysis showed that inflammatory pathways were the most frequently implicated, including “cytokine-cytokine receptor interaction,” “chemotaxis,” and “GPCR ligand binding” (Figure 2A). Moreover, these genes were gathered in 10 MCODE components, including “NABA secreted factors,” “cytokine-cytokine receptor interaction,” and “NABA matrisome associated” (Figure 2B and Supplementary Table 3).

### Identification of the Predictive Six-IRG Signature

**FIGURE 3:**
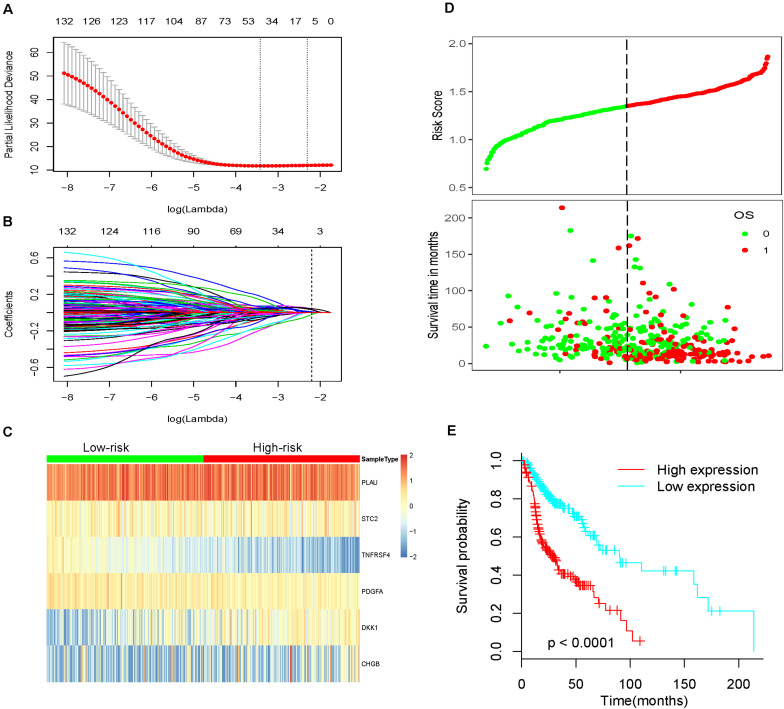
Construction of the most valuable prognostic IRG signature by using a LASSO Cox model. **(A)** The LASSO coefficient profiles of six IRGs; a vertical line is drawn at the value identified by 10-fold cross-validation. **(B)** Tuning parameter (Lambda, λ) selection cross-validation error curve. Vertical lines were drawn at the optimal values given by the minimum criteria and 1-SE criteria. The right line was identified by 1-SE criteria (SE = 0.094, λ = 0.028). **(C)** Heatmap showing the prognostic six-IRG signature in the training cohort. **(D)** The distribution of risk score, overall survival time, and OS status. The dotted line indicates the cut-off point for the optimal risk score used to stratify patients into low-risk and high-risk groups. **(E)** Survival analysis based on the six-IRG signature in the training cohort. The survival curve shows that the patients in the high-risk group had worse prognosis outcomes.

We obtained survival-associated data from the TCGA-HNSCC dataset. To avoid interference due to unrelated causes of death, patients with a follow-up time < 30 days were excluded. Next, we performed univariable Cox regression analysis for 339 differentially expressed IRGs and survival data from 492 patients; 136 prognosis-related IRGs were identified as *p* < 0.05. Then, all 492 patients were randomly divided into a training cohort and a testing cohort in a 3:1 ratio. The training cohort was used to precisely construct a predictive model for the survival of HNSCC patients. Next, we performed LASSO Cox regression to establish the most optimal prognostic IRG signature for HNSCC patients (Figures 3A,B). As a result, a prognostic IRG signature was established, consisting of six immune-related genes (PLAU, STC2, TNFRSF4, PDGFA, DKK1, CHGB). The formula used to calculate the risk score of the six-IRG signature was as follows: Risk score = PLAU^∗^0.0496 +STC2^∗^0.0393-TNFRSF4^∗^0.0405+PDGFA^∗^0.00743+DKK1^∗^ 0.0482+CHGB^∗^0.0147. Then, all patients were separated into a high-risk group and a low-risk group based on the median expression of the six-IRG signature. The risk score distributions of the six-IRG signature, survival status, and the expression profile of the six IRGs are shown in Figures 3C,D. Patients in high-risk groups showed poorer prognoses, while patients in low-risk groups had more favorable prognoses. Kaplan–Meier survival analysis revealed that patients in the high-risk group were associated with a trend toward worse survival compared with patients in the low-risk group (Figure 3E).

### Correlation of the Six-IRG Signature With the Clinicopathological Characteristics of Patients

To investigate the correlation between the six-IRG signature and clinicopathological data, we collected a range of data from the TCGA database, including patient age, gender, tumor grade, and tumor stage. All patients were assigned to the high-risk and low-risk groups based on the cut-off value of the six-IRG signature. The association between the six-IRG signature and clinicopathological characteristics was then analyzed for patients with HNSCC. As shown in Table 1, the risk score for the six-IRG signature showed a significant association with tumor stage. However, there was no association between risk score and age, gender, or the tumor grade of patients.

**TABLE 1:** Association of six-IRG signature with clinicopathological characteristics.

Variable	Total patients	Risk score		
		
	No (%)	Low-risk group	High-risk group	*p*
**Age**				
Mean ± SD (years)	60.77 ± 11.61	61.04 ± 11.85	60.49 ± 11.39	0.65
**Age**				0.16
≤65	102 (27.6)	45 (44.1)	57 (55.9)	
=65	267 (72.4)	140 (52.4)	127 (47.6)	
**Gender**				0.53
Male	277 (71.6)	139 (81.1)	138 (18.9)	
Female	92 (28.4)	46 (73.3)	46 (26.7)	
**Stage**				0.005
I	19 (5.1)	13 (68.4)	6 (31.6)	
II	61 (16.5)	37 (60.7)	24 (39.3)	
III	70 (19.0)	37 (52.9)	33 (47.1)	
IV	219 (59.3)	98 (44.7)	121 (55.3)	
**Grade**				0.62
Low	265 (74.4)	128 (48.3)	137 (51.7)	
High	91 (25.6)	49 (53.8)	42 (46.2)	
**HPV**				0.0069s
Negative	50 (75.8)	26 (52.0)	24 (48.0)	
Positive	16 (24.2)	15 (93.8)	1 (6.2)	

### Validation of the Six-IRG Signature as an Independent Prognostic Factor

To explore whether the six-IRG signature was a clinically independent prognostic factor for HNSCC patients, we performed univariable and multivariable Cox regression analyses. The risk score of the six-IRG signature and other clinicopathological data, such as age, gender, tumor grade, and tumor stage, were included as covariates. Only covariates that were significant in univariable Cox regression were used in multivariable Cox regression. As shown in Table 2, the six-IRG signature remained as an independent prognostic factor even after adjustment for stage and other prognostic factors in the multivariable analyses.

**TABLE 2:** Univariable and multivariable Cox regression analyses of six-IRG signature.

Variable	Univariable Cox regression			Multivariable Cox regression		
		
	OR	95%CI	*p*	OR	95%CI	*p*
Gender	1.259	0.89–1.77	0.2			
Age (55/56)	1.22	0.84–1.77	0.3			
Stage	1.37	1.12–1.66	0.002	1.25	1.03–1.53	0.028
Grade	1.051	0.83–1.33	0.7			
Six-IRG signature	23.09	9.90–53.84	2e-13	19.82	8.48–46.31	5.38e-12

### Subgroup Analysis of the Six-IRG Signature for Survival Prediction

**FIGURE 4:**
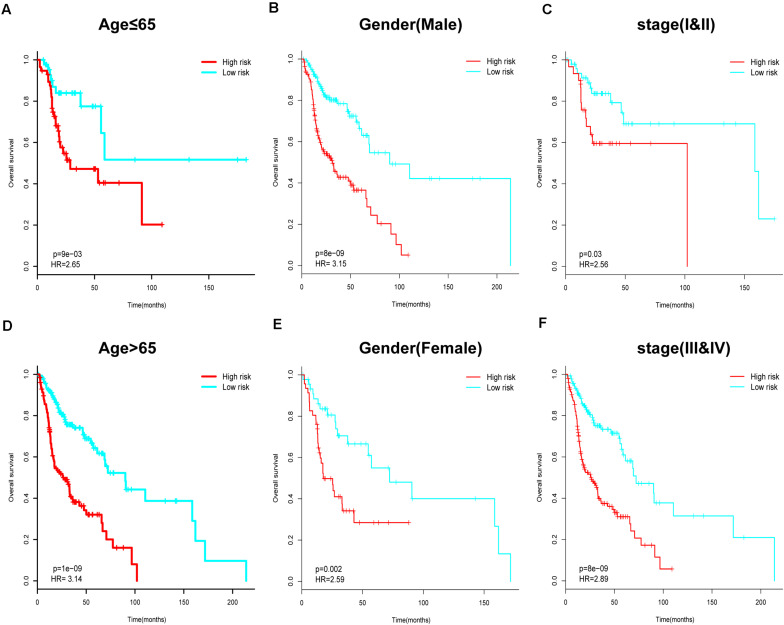
Subgroup analysis for the six-IRG signature when predicting survival by stratifying patients with various clinicopathological risk factors. The patients were stratified into six subgroups for survival analysis based on **(A)** age > 65, **(B)** age ≤ 65, **(C)** gender (male), **(D)** gender (female), **(E)** stage I and II, and **(F)** stage III and IV.

Next, we performed further subgroup analysis to evaluate the prognostic value of the six-IRG signature within the same clinicopathological risk factors. The patients were assigned into different subgroups, including a younger group (age ≤ 65 years), an elder group (age > 65 years), a male group, a female group, an earlier-stage group, and an advanced-stage group. Subgroup analysis revealed that the six-IRG signature could still distinguish patients into different survival groups within all subgroups with statistically significant prognostic value (Figures 4A–F).

### Validation of the Six-IRG Signature for Survival Prediction

**FIGURE 5:**
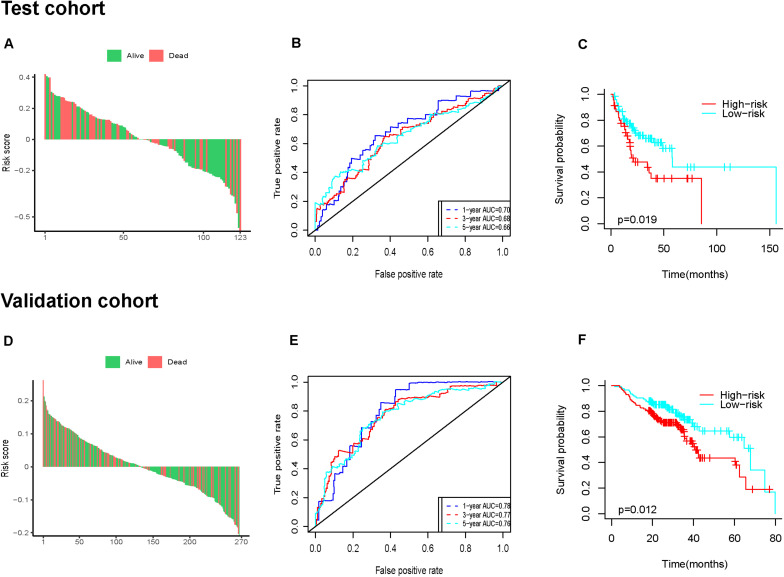
Prognostic analysis of the six-IRG signature in the test cohort and validation cohort. **(A,D)** The distributions of risk scores in the test cohort and validation cohort, respectively, **(B,E)** the survival ROC curves in the test cohort and validation cohort, respectively, **(C,F)** Kaplan–Meier survival curves between the high-risk group and low-risk group in the test cohort and validation cohort, respectively. *p*-value was determined by the log-rank test.

Next, we further assessed the prognostic value of the six-IRG signature in the test cohort and the validation cohort (Figures 5A,B). Patients were divided into a high-risk group and a low-risk group using the median cut-off value for risk score, which was used in each cohort separately. The results of the survival analysis demonstrated that the six-IRG signature was a favorable prognostic marker (Figures 5A,D). The survival status of patients in the low-risk group was more favorable, while those in the high-risk group had poorer survival (Figures 5C,F). Receiver operator characteristic (ROC) curves indicated that the six-IRG signature performed with good prognostic accuracy in both the test cohort and the validation cohort (Figures 5B,E).

### Establishment of a Predictive Nomogram Model Based on the Six-IRG Signature

**FIGURE 6:**
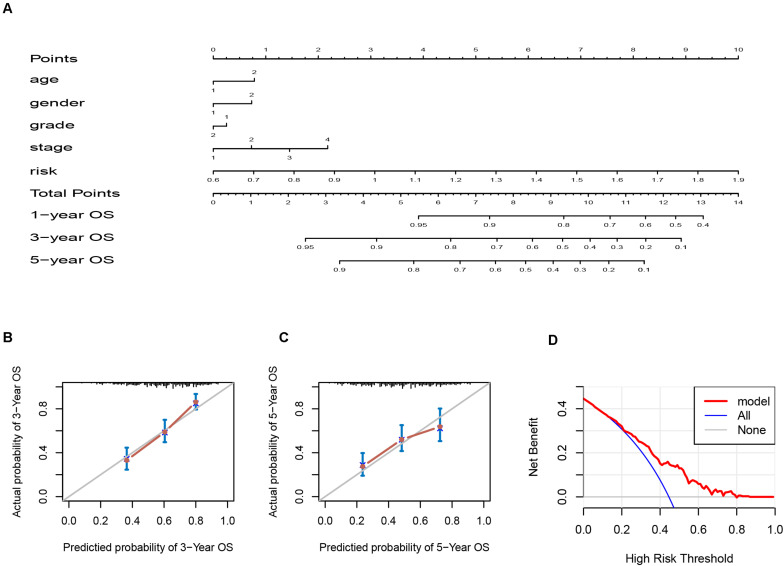
Nomogram and calibration plots for the prediction of survival for patients with HNSCC. **(A)** Nomogram for the prediction of OS at 1, 3, and 5 years. **(B,C)** Calibration plots for predicting OS at 3 and 5 years, Diagonal line: ideal model, vertical bars: 95% confidence interval. **(D)** DCA for assessing the clinical utility of the nomogram. The *x*-axis indicates the percentage of threshold probability while the *y*-axis indicates the net benefit.

Since the six-IRG signature exhibited a strong prognostic ability, we next tried to establish a novel nomogram model to predict the clinical prognosis of patients with HNSCC by integrating the six-IRG signature with other prognostic factors, including age, gender, tumor grade, and tumor stage. Based on a total number of points in the nomogram for all prognostic factors, we could provide individualized risk prediction for HNSCC patients in an accurate manner (Figure 6A). Calibration plots were used to estimate the predictive value of the nomogram, results showed that the nomogram exhibited a favorable predictive value when compared with an ideal model (Figures 6B,C). Moreover, decision curve analysis (DCA) indicated that the nomogram had a high potential for clinical utility (Figure 6D).

### Correlation Analysis Between the IRG Signature and Immune Cell Infiltration

**FIGURE 7:**
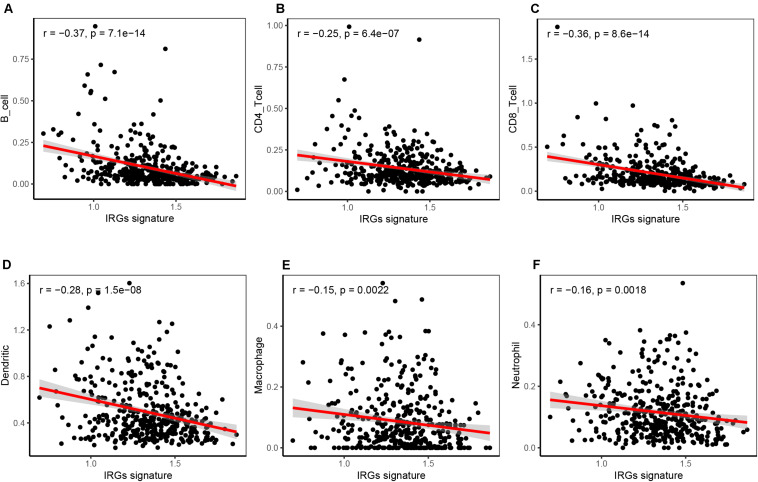
Correlation analysis showing that the six-IRG signature was significantly associated with immune cell infiltration in the TCGA HNSCC dataset. **(A)** The six-IRG signature was negatively correlated with B cell infiltration, **(B)** CD4 T cell infiltration, **(C)** CD8 T cells infiltration, **(D)** dendritic cell infiltration, **(E)** macrophage infiltration, and **(F)** neutrophil infiltration in the HNSCC patient cohort based on the TCGA database. The significance was determined by Pearson’s correlation coefficient.

The tumor immune microenvironment consisted of massive immune cell subsets surrounding cancer cells, including B cells, CD4^+^ T cells, CD8^+^ T cells, neutrophils, macrophages, and dendritic cells. We employed the TIMER web portal to estimate the tumor purity and the abundance of infiltrating immune cells based on the HNSCC datasets, and performed correlation analysis between the immune cell infiltration and the six-IRG-based signature expression. Notably, the expression value of each IRG gene and six-IRG-based signature showed a significantly negatively association with B cells, CD4^+^ T cells, CD8^+^ T cells, neutrophils, macrophages, and dendritic cells (Figures 7A–F and Supplementary Figure 1).

### Genetical Alteration and Interaction Network for the Six IRGs

**FIGURE 8:**
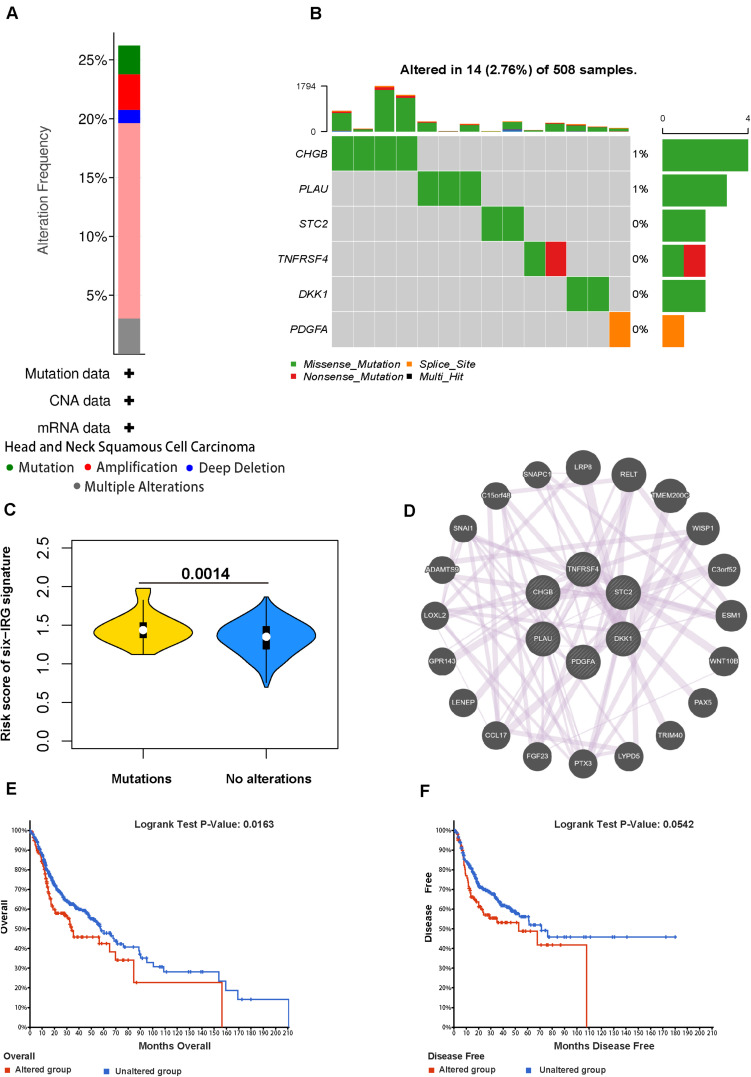
Genetic alterations of the six IRGs in HNSCC. **(A)** The total frequency of genomic alterations for the six immune-related genes in the HNSCC dataset based on the TCGA database. **(B)** A visual summary (Oncoprint plot) of genomic alterations located in the genomic region of the six IRGs. **(C)** The comparison of the risk score in mutant patients and non-mutant patients. **(D)** The interaction network for the six immune-related genes. **(E,F)** The overall survival and disease-free survival analyses of the genomic altered group and the unaltered group based on the six immune-related genes.

We analyzed the genetic status of the six IRGs; these six IRGs showed genetic alteration in 139 out of 530 cases (Figure 8A). Then we used the “oncoplot” program in the maftools package to identify mutations in the six IRGs (Figure 8B). CHGB and PLAU showed the most alterations, the most common alteration was missense mutation. The IRG scores in patients with mutations were significantly higher compared with those with no alterations (Figure 8C). Kaplan-Meier survival analysis indicated that the overall survival (OS) and disease-free survival (DFS) of patients with genetic alterations were poorer (*p* < 0.05) than those in the unaltered group (Figures 8E,F). Next, we used GeneMANIA to identify an interaction network for the six IRG genes; this analysis indicated that there were numerous interactions and co-expression profiles among the six genes and other immune-related genes, and that the pathways were involved in multiple immune-related networks (Figure 8D).

### Validating the Expression of the Six IRGs in HNSCC

**FIGURE 9:**
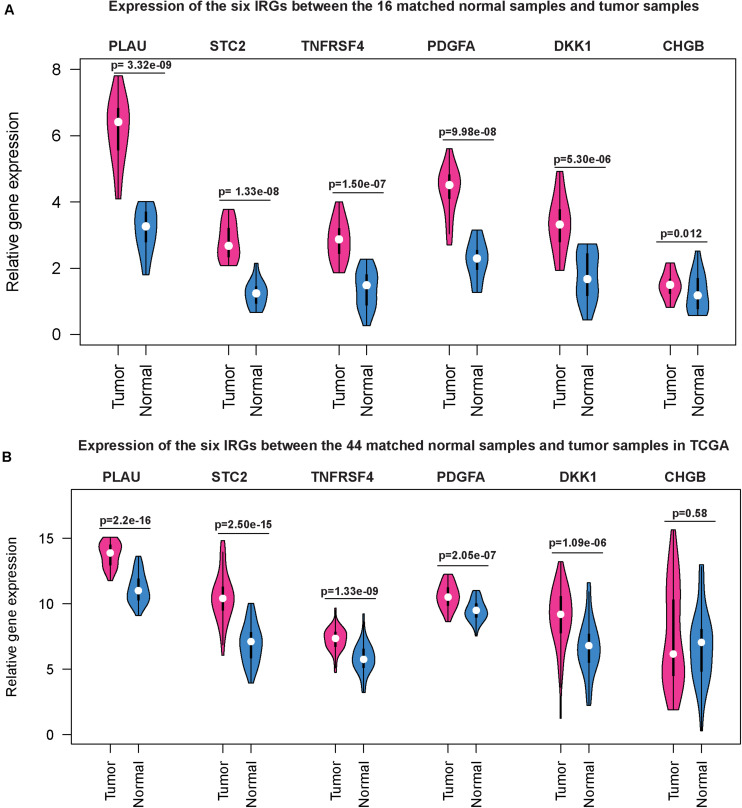
Gene expression level of the six IRGs in patients with HNSCC. qRT-PCR results showed that the mRNA expression levels of the six IRGs were all significantly higher (*P* < 0.05) in HNSCC tissues than those in adjacent normal tissues **(A)** and the same trend was observed in the HNSCC dataset based on the TCGA database **(B)**.

Next, we used qRT-PCR to detect the expression levels of the six IRGs in HNSCC and paired normal tissues. Compared with the paired normal group, the relative mRNA levels of PLAU, STC2, TNFRSF4, PDGFA, and DKK1 were significantly higher (*p* < 0.05) in HNSCC tissues than those in adjacent normal tissues (Figure 9A), the results were very similar to the corresponding expression differences between the 44 matched normal samples and tumor samples in TCGA (Figure 9B), except that the CHGB gene was overexpressed in the validation assay but had relatively low expression in the HNSCC sample of TCGA.

### Comparison With Other HNSCC Prognostic Models

To evaluate the predictive capability of the six-IRG signature for clinical use in the HNSCC patient cohort, we compared our prognosis model with the other two published immune-score-based clinical prognosis models. Qiu’s model consisted of 11 immune-related genes, including TGFB1, MMP9, PLAU, CTSG, CCR8, SEMA5B, GAST, OSM, IL12RB2, TNFRSF25, and TNFRSF4, and showed a predictive accuracy with a C-index of 0.732 ± 0.02 and an AIC of 1726.89 (Qiu et al., 2020); She and colleagues (2020) constructed a predictive model based on 27 immune-related gene signatures with a C-index of 0.746 ± 0.005 and an AIC of 1689.87. The results showed that our model had a very similar sensitivity and accuracy when compared with the other models (Supplementary Table 4).

## Discussion

An increasing number of recent research studies have indicated that immune regulation plays a key role in carcinogenesis and progression, and was associated with the clinical outcome and therapeutic responsiveness of HNSCC (Zhang et al., 2018; Oliva et al., 2019; Wang et al., 2019a,b). Zhang et al. (2018) found that CD3^+^ and CD8^+^ cell infiltration was correlated with the progression of HNSCC. And the immune-score based on CD3^+^ and CD8^+^ cell infiltration could be a useful prognostic marker. Schneider and his team also found that high CD3^+^ T-lymphocytes infiltration was correlated with a significantly better overall survival of HNSCC (Schneider et al., 2018). She et al. (2020) identified a prognostic signature including 27 immune-related genes that were associated with the overall survival of HNSCC patients and could be used as a favorable predict marker. A retrospective study indicated that increased tumoral infiltration by CD8^+^ T cells and an increased ratio of CD8^+^ T cells/Tregs were positively correlated with treatment response to anti-PD-1/PD-L1 agents (Hanna et al., 2018). Hence, we also analyzed the relationship of the IRGs signature of our prognostic model and immune cell infiltration, the result demonstrated that the six-IRG signature was significantly associated with immune cell infiltration.

In this study, we performed a comprehensive bioinformatics analysis to explore the potential prognostic value of immune-related genes. First, we used RNA-seq data of HNSCC tumors from the TCGA database to identify 439 differentially expressed IRGs. Then according to biological pathway enrichment analysis, we found that inflammatory pathways were most frequently implicated, including cytokine-cytokine receptor interaction, chemotaxis, and GPCR ligand binding, etc. In previous studies, these inflammatory pathways were usually found to be activated with the progression of HNSCC, and the persistent expression of key genes in this inflammatory pathway caused cancer cell proliferation, invasion, and metastasis, and the low survival rate of HNSCC patients (Thurlow et al., 2010; Li et al., 2020b).

To explore whether immune-related genes can be used as biomarkers in predicting the survival time of HNSCC patients, we performed a Cox regression analysis to identify prognosis-related genes from differentially expressed immune-related genes. The 136 IRGs showed a favorable prognostic value and were used to construct a prognostic gene signature. LASSO Cox regression is an optimal method for high dimensional datasets to construct a prognostic signature. According to LASSO regression analysis, we obtained a prognostic IRGs signature consisting of six immune-related genes, including PLAU, STC2, TNFRSF4, PDGFA, DKK1, and CHGB. Previous studies have established the involvement of these six immune-related genes in invasion and metastasis of HNSCC tumor cells. More recently, both PLAU and STC2 were identified as oncogenes in HNSCC tumors, associated with immunosuppression and inflammation (Chang et al., 2003; Ieta et al., 2009; Na et al., 2015; Yang et al., 2017, 2020; Joshi, 2020; Fang et al., 2021; Li et al., 2021). TNFRSF4 (also known as CD134/OX40) is one of the representative targets of second-generation immune checkpoints, and its clinical efficacy has been observed by anti-TNFRSF4 (MEDI6469) therapy in HNSCC patients through regulating antigen-specific tumor-infiltrating T cells (Duhen et al., 2021). PDGFA belongs to the platelet-derived growth factor protein family, which regulates the tumor microenvironment of HNSCC through the PDGF/AKT signaling pathway (Duhen et al., 2021). DKK1 is well known for its roles in the regulation of the tumor microenvironment and immune response by the Wnt pathway, and has been reported as an independent unfavorable prognostic indicator of HNSCC survival (Gao et al., 2018; Haas et al., 2020). Aberrant expression of the CHGB gene has been reported in various tumors, and its upregulated expression is highly correlated with metastasis (Chen et al., 2020b; Xu et al., 2020). Previous research demonstrated that CHGB promoted the occurrence and advanced lymph node metastasis of nasopharyngeal carcinoma (Zhou et al., 2007).

Furthermore, we analyzed the reliability and stability of this prognostic model consisting of six immune-related genes. The results of univariable and multivariable Cox regression analyses showed that the six-IRG signature could serve as a clinically independent prognostic factor in HNSCC patients. Then, we developed a nomogram combining the six-IRG signature with other clinical characteristics. We also explored the correlation of the six-IRG signature with patients’ clinical variables. We found that the six-IRG signature showed a positive association with the tumor stage. Therefore, our prognostic model showed high clinical applicability in predicting the prognosis of HNSCC. Compared to other current clinical tools (Qiu et al., 2020; She et al., 2020) available for prognosis including the immunoscore, our model showed similar sensitivity and accuracy.

Inevitably, there were some limitations in this study. First, in our study, we tried to obtain as many HNSCC cohorts as possible to build a more sufficient dataset to validate our prognostic signature. However, the result was not validated in prospective clinical trials. Second, some important clinical information was not available for public access. For example, we did not know the patient’s infection status including whether they used anti-inflammatory drugs, which may bias the experimental results. Third, the biological or medical mechanisms behind identified IRGs were not very clear, and more experimental research studies are needed on the tumor microenvironment to provide more important information for further understanding of their functional roles in the HNSCC.

## Conclusion

In conclusion, our research provided a novel immune-related genes signature to estimate prognosis for HNSCC patient survival. The IRGs signature was valuable for its association with immune infiltrate levels. The correlation of the IRGs with overall survival in the comprehensive collection of patient cohorts indicated that it was a powerful prognostic marker for HNSCC, and might facilitate HNSCC patient’s counseling and individualized management.

## Data Availability Statement

The original contributions presented in the study are included in the article/Supplementary Material, further inquiries can be directed to the corresponding author/s.

## Ethics Statement

The studies involving human participants were reviewed and approved by the Renmin Hospital of Wuhan University. The patients/participants provided their written informed consent to participate in this study.

## Author Contributions

JW and YZ conceived and designed the study. JW, QZ, QH, and HZ performed the analysis procedures. JW, YZ, QZ, QH, and HW analyzed the results. YZ, QZ, and PC contributed to the analysis tools. JW, YZ, HW, and HZ contributed to the writing of the manuscript. All authors reviewed the manuscript.

## Conflict of Interest

The authors declare that the research was conducted in the absence of any commercial or financial relationships that could be construed as a potential conflict of interest.

## Footnotes

1 cistrome.shinyapps.io/timer

2 http://cbioportal.org

## Supplementary Material

Supplementary Figure 1 Correlation analyses showing that all six-IRG showed significant correlations with the infiltrationof various types of immune cell.

## Abbreviations

DCA: Decision curve analysis
DEGs: Differentially expressed genes
GO: Gene Ontology
HNSCC: Head and neck squamous cell carcinoma
HPV: Human papillomavirus
IRGs: Immune-related genes
LASSO: Least absolute shrinkage and selection operator
OS: Overall survival
PPI: Protein-protein interaction
TCGA: The Cancer Genome Atlas
TIICs: Tumor-infiltrating immune cells
ROC: Receiver operating characteristic curve
SNP: Single nucleotide polymorphisms.
