# Supplementary figures and images for: A Novel Immune-Related Prognostic Signature in Head and Neck Squamous Cell Carcinoma
Source: Front Genet. 2021 Jun 18;12:570336. doi: 10.3389/fgene.2021.570336 (PMC8249947; doi:10.3389/fgene.2021.570336)

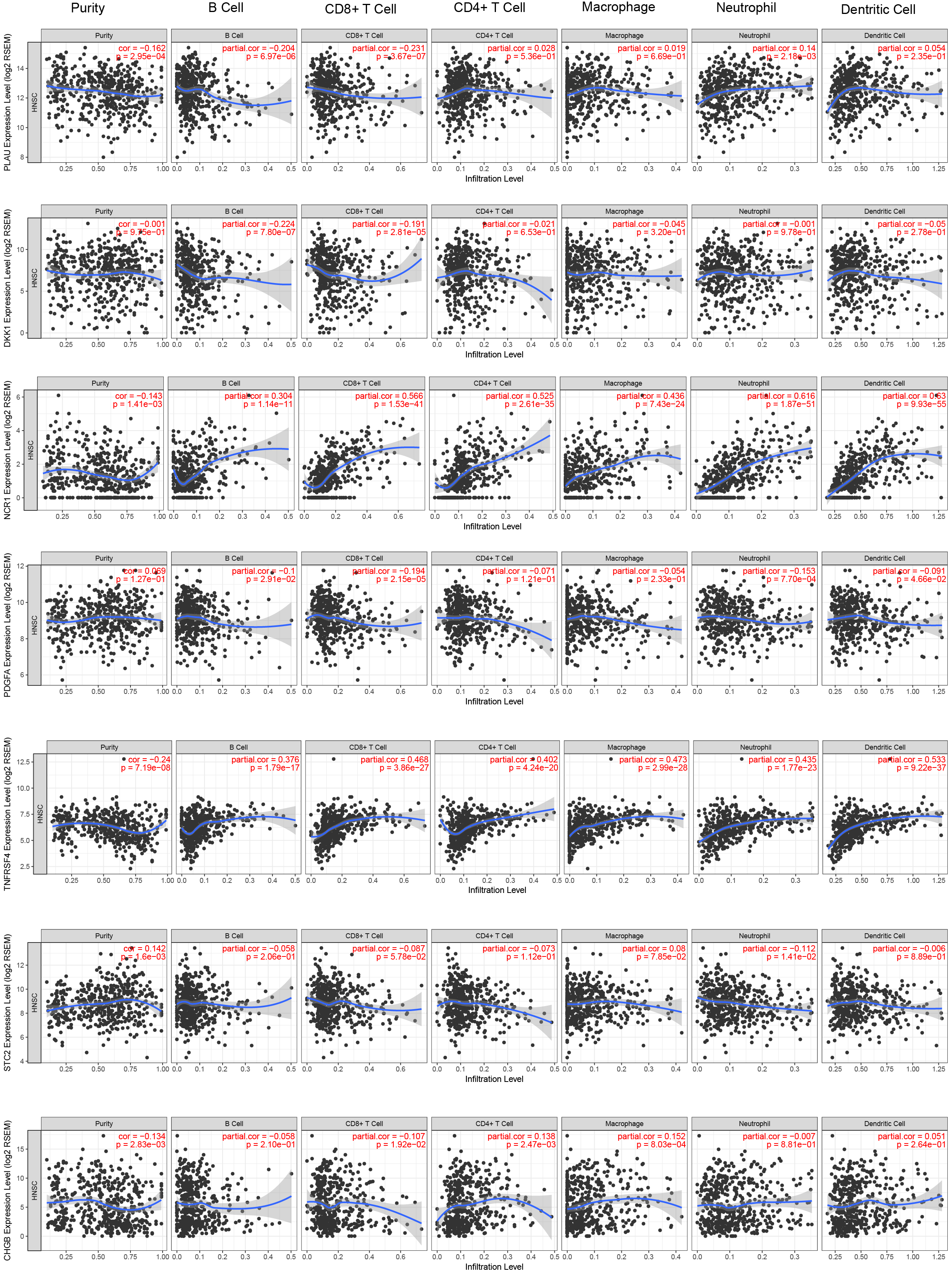

Supplement: Supplementary Figure 1 — Correlation analyses showing that all six-IRG showed significant correlations with the infiltrationof various types of immune cell. [file Image_1.tif]
