# Supplementary material for: A Novel Immune-Related Prognostic Signature in Head and Neck Squamous Cell Carcinoma
Source: Front Genet. 2021 Jun 18;12:570336. doi: 10.3389/fgene.2021.570336 (PMC8249947; doi:10.3389/fgene.2021.570336)
Supplement: Supplementary file 3 [file Table_2.docx]

**Supplementary Table 2**

The primers used for qRT-PCR to amplify the six-IRG signature from HNSCC patients.

| **Genes** | | **Genes ID** | **Sequence** | |
| --- | --- | --- | --- | --- |
| CHGB Forward primer | | NG_042285.1 | TCAGCATGGCCAGTTTAGGG | |
| CHGB Reverse primer | |  | CTCTGTGATGGCGTCCCTTT | |
| STC2 Forward primer | | NM_003714.2 | TGTCCCTGCAGAATACAGCG | |
| STC2 Reverse primer | |  | AGCGTGGGCCTTACATTTCA | |
| DKK1 Forward primer | | NM_012242.4 | TGGAACTCCCCTGTGATTGC | |
| DKK1 Reverse primer | |  | AATAGGCAGTGCAGCACCTT | |
| PLAU Forward primer | | NG_011904.1 | CCAGGGTCCACCTGTCCC | |
| PLAU Reverse primer | |  | CTTCATCTCCCCTTGCGTGT | |
| TNF Forward primer | | NG_007462.1 | CGACAGCAGAGACGAGGATG | |
| TNF Reverse primer | |  | CCTGTCCTCACAGATTGCGT | |
| PDGFA Forward primer | | NG_029727.1 | GGTCGCTCCTGAAGCCAG | |
| PDGFA Reverse primer | |  | GGAGGAGAAACAGGGAGTGC | |
| GAPDH Forward primer | | NM_001115114.1 | | GCACCACCAACTGCTTAGCA |
| GAPDH Reverse primer | |  |  | GTCTTCTGGGTGGCAGTGATG |
