# Supplementary material for: A Novel Immune-Related Prognostic Signature in Head and Neck Squamous Cell Carcinoma
Source: Front Genet. 2021 Jun 18;12:570336. doi: 10.3389/fgene.2021.570336 (PMC8249947; doi:10.3389/fgene.2021.570336)
Supplement: Supplementary file 5 [file Table_4.docx]

**Supplementary Table 4**

**Comparison with other HNSCC prognostic models**

| Model | Overall survival | |
| --- | --- | --- |
|  | C-index | AIC |
| Our model | 0.732±0.02 | 1726.89 |
| Qiu’s model^[1]^ | 0.725±0.04 | 1786.34 |
| She’s model^[2]^ | 0.746±0.005 | 1689.87 |

Reference List

1. Qiu Y, Cui L, Lin Y, Gao B, Li J, Zhao X, et al. Development and Validation of a Robust Immune Prognostic Signature for Head and Neck Squamous Cell Carcinoma. Front Oncol. 2020;10:1502. Epub 2020/11/24. doi: 10.3389/fonc.2020.01502. PubMed PMID: 33224866; PubMed Central PMCID: PMCPMC7667274.

2. She Y, Kong X, Ge Y, Yin P, Liu Z, Chen J, et al. Immune-related gene signature for predicting the prognosis of head and neck squamous cell carcinoma. Cancer Cell Int. 2020;20:22. Epub 2020/01/29. doi: 10.1186/s12935-020-1104-7. PubMed PMID: 31988638; PubMed Central PMCID: PMCPMC6969412.
